# Contemporary Definitions of Infant Growth Failure and Neurodevelopmental and Behavioral Outcomes in Extremely Premature Infants at Two Years of Age

**DOI:** 10.21203/rs.3.rs-3228742/v1

**Published:** 2023-08-09

**Authors:** Katie Strobel, Thomas Wood, Gregory Valentine, Kendell German, Semsa Gogcu, D Hendrixson, Sarah Kolnik, Janessa Law, Dennis Mayock, Bryan Comstock, Patrick Heagerty, Sandra Juul

**Affiliations:** University of Washington; University of Washington; University of Washington; University of Washington; Wake Forest; University of Washington - Seattle Children's Hospital; University of Washington; University of Washington

## Abstract

**Introduction:**

The association of 2-year neurodevelopmental and behavioral outcomes with in-hospital or post-discharge growth failure (GF) using contemporary definitions for preterm infants is unknown.

**Methods:**

In a secondary analysis of a preterm cohort, changes in anthropometric z-scores were examined between birth and hospital discharge, and from discharge to 2 years. The 2-year evaluation included Bayley Scales of Infant Development (BSID-III) and Child Behavior Checklist (CBCL).

**Results:**

Among 629 infants, accelerated linear growth from birth to discharge was associated with higher BSID-III cognitive scores (+ 3.2 points [IQR 0.02, 6.4]) while in-hospital GF was not associated with any outcomes. Infants with weight GF after discharge had lower BSID-III motor scores (−3.1 points [−5.9, −0.2]). Infants with accelerated weight growth after discharge had increased odds of behavioral problems on the CBCL (aOR 1.9 [1.03, 3.5]).

**Discussion:**

In-hospital and post-hospitalization growth metrics are modestly associated with neurodevelopmental outcomes with length gains apparently most beneficial.

## INTRODUCTION

Providing adequate nutrition to the premature infant for the first two years is essential for neurodevelopment, the gut microbiome, bone health, and metabolism (1-4). The most common markers of adequate nutritional status are serial measurements of anthropometrics (weight, length, and head circumference). However, the definition of inadequate growth, or “growth failure,” during the neonatal intensive care unit (NICU) admission is controversial (5). Furthermore, there is limited information on what is optimal growth once the infant is discharged from the NICU.

Extremely preterm infants are at particularly high risk for both growth failure and neurodevelopmental impairment. Early studies examining growth failure in extremely low birth weight (ELBW) infants defined growth failure based on changes in growth velocity and small for gestational age status at 36 weeks postmenstrual age (PMA) (6-8). Greater weight gain was associated with improved Bayley Scales of Infant Development (BSID)-II mental and psychomotor subscales at 18 months (6, 8) and at 22 months (6). Similar findings were found for head circumference growth (6, 8). However, increased weight gain or “catch up growth,” has also been associated with lower lean body mass and increased total body fat in adolescence (9). Catch up growth in infancy has additionally been associated with later obesity and increased risk for metabolic diseases (10). Studies utilizing growth metrics such as growth velocity and small for gestational age status at 36 weeks are limited because they did not take into account the infant’s initial growth percentiles.

To address this concern, many groups now consider changes in weight or length z-score as a better measure for adequate growth (2, 11-13). Studies of the relationship between changes in z-score during the initial hospitalization of ELBWs and neurodevelopmental outcomes show mixed results (8, 14, 15), and there has been minimal research evaluating the association of z-score changes after discharge with neurodevelopment (16).

The objective of this study was to examine changes in weight, length, and head circumference z-scores from birth to discharge and from discharge to two years and their associations with neurodevelopmental and behavioral outcomes. We hypothesized that poor linear growth would be associated with adverse neurodevelopmental and behavioral outcomes at two years of age.

## METHODS

### Patient Population:

This study is a secondary analysis of the Preterm Erythropoietin Neuroprotection (PENUT) Trial (NCT #01378273) (17). PENUT was a randomized, double-blinded, placebo-controlled trial of erythropoietin for neuroprotection in infants born 24 through 27 completed weeks of gestation. The study was conducted at 19 sites and 30 neonatal intensive care units in the United States between December 2013 and September 2016 (17). All infants enrolled in the PENUT trial who survived and were assessed for long-term developmental outcomes were eligible for this study excepting those requiring invasive mechanical ventilation at discharge, as their growth may be particularly disrupted (N = 17) (17). The PENUT Trial was approved by an institutional review board at each site. Parental consent was obtained prior to infant enrollment. This study followed the Strengthening the Reporting of Observational Studies in Epidemiology (STROBE) and Transparent Reporting of a multivariable prediction model for Individual Prognosis or Diagnosis (TRIPOD) reporting guidelines.

We collected data about maternal characteristics, pregnancy, and delivery, as well as infant characteristics including anthropometric measurements, time to regain birth weight, exposure to medications, and comorbidities during their NICU stay (17). At 20 to 33 months corrected age (CA), infants were evaluated by certified examiners who assessed cognitive, motor, and language development with the BSID-lll. All BSID-III subscales were scaled based on the age at which the assessment was performed. Child Behavior Checklist (CBCL) for ages 1–5 years was administered, and T scores for emotionally reactive, anxious/depressed, somatic complaints, withdrawn, sleep problems, attention problems, aggressive behavior problems, depressive problems, anxiety problems, autism spectrum problems, attention deficit/hyperactivity problems, oppositional defiant problems, and total problem score were collected. The Modified Checklist for Autism in Toddlers (M-CHAT-R) was also given, and the total score was collected.

### Growth Parameters Analyzed:

#### Birth to Discharge.

Weight, length, and head circumference were collected at birth, 14 days, and discharge. Fenton growth curves were utilized to calculate weight, length, and head circumference z-scores for growth parameters during the NICU hospitalization (18). Weight, length, and head circumference growth failure were defined as: z-score discharge – z-score birth ≤−0.8, as defined by the Academy of Nutrition and Dietetics (13). Weight, length, or head circumference accelerated growth was defined as: z-score discharge – z-score birth ≥0.8 for weight or length. Normal growth was defined as a z-score change of −0.799 to 0.799. If z-score changes were >3 or <−3, they were truncated to 3 or −3 to avoid bias in likely input errors.

#### Discharge to two years.

Weight, length, and head circumference were measured at two years corrected age (CA), and body mass index (BMI) z-scores were calculated using Centers for Disease Control and Prevention (CDC) growth curves (19). Changes in weight, length, and head circumference z-score were assessed from discharge to two years CA. Weight, length, or head circumference growth failure were defined as: z-score 2 years – z-score discharge ≤−0.8 (13). Weight or length accelerated growth was defined as: z-score 2 years – z-score discharge ≥0.8. Normal growth was defined as a z-score change of −0.799 to 0.799. If z-score changes were >3 or <−3, they were truncated to 3 or −3 to avoid bias in likely input errors.

### Statistical Analyses:

Summary demographic variables are presented as mean and standard deviation (SD) except for days to regain birthweight which is presented as median with interquartile range (IQR). Z-score calculations were completed using the peditools library in R, and the cohorts were divided into growth failure, normal growth, and accelerated growth for weight, length, and head circumference growth. For all inferential analyses, generalized estimating equations (GEE) with robust standard errors were used to appropriately account for potential correlation of outcomes for same-birth siblings (20). Baseline and demographic factors were compared across growth trajectory groups using a multivariate Wald test.

Linear GEE models adjusted for gestational age, maternal education, > 14 days of dexamethasone, length z-score at birth, severe intracranial hemorrhage (grade III or IV) (ICH), erythropoetin use, and feeding status at discharge were used to compare growth categories with respect to neurodevelopmental scores (BSID-III and M-CHAT-R). GEE logistic regression models were used to determine adjusted odds ratios (aOR) for having a borderline/clinical score in CBCL sub-parameters based on post-discharge growth trajectory, adjusting for gestational age, maternal education, > 14 days of dexamethasone, length z-score at birth, severe ICH, erythropoetin use, and feeding status at discharge. Model outputs are presented as adjusted mean difference or aOR with 95% confidence intervals (CI). All analyses were conducted using R statistical package (Version 4.1.2, Foundation for Statistical Computing, Vienna, Austria). A p-value < 0.05 was considered statistically significant.

## RESULTS

### Growth from Birth to NICU Discharge

Six hundred and twenty-nine infants met criteria for this secondary analysis. Maternal and child characteristics for infants with length growth failure, normal growth, and accelerated growth are shown in Table 1. Maternal and child characteristics for infants with weight growth failure, normal growth, and accelerated growth are shown in **Supplementary Table 1**. Common co-morbidities of prematurity including necrotizing enterocolitis (stage 2b or 3), severe ICH, severe bronchopulmonary dysplasia (BPD), symptomatic culture positive sepsis, and length of stay are included. The linear growth failure group had more mothers with a high school education or less and infants born at a lower gestational age (Table 1). The weight growth failure group were less likely to be small for gestational age at birth (**Supplementary Table 1**).

BMI z-score at two years did not differ between those with weight or length accelerated, normal growth, or growth failure during their initial hospitalization. Growth velocity in the weight failure group was 19.6 (3.5) g/d compared to 24.0 (4.2) and 27.0 (4.1) in normal and accelerated growth cohorts, respectively (p<0.001) (**Supplementary Table 1**). Similarly, increase in length was slower in the length growth failure group compared to normal and accelerated growth cohorts: 0.91 (0.2) cm/week, 1.1 (0.1), and 1.5 (0.2) in growth failure, normal, and accelerated growth cohorts, respectively (p<0.001) (Table 1).

**Figure 1:**
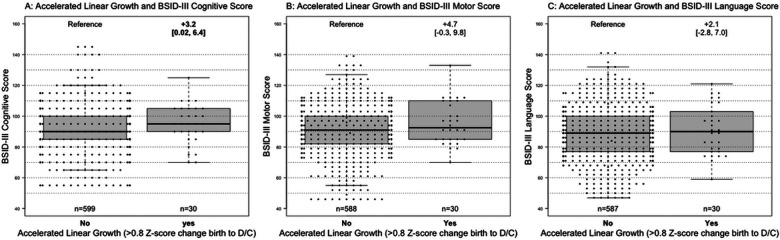
A-C: Accelerated linear growth from birth to discharge (D/C) and Bayley Scales of Infant Development III (BSID-III) scores. Boxplots of BSID-III cognitive, motor, and language scores comparing infants who experienced accelerated linear growth from birth to discharge to those that did not, adjusting for gestational age, maternal education, >14 days of dexamethasone, erythropoietin use, length z-score at birth, pregnancy induced hypertension, severe bronchopulmonary dysplasia, and grade 3-4 intracranial hemorrhage. Estimated mean difference in the accelerate growth group with 95% CI is presented above the boxplot, with significant differences in bold.

After adjusting for gestational age, maternal education, >14 days of dexamethasone, erythropoietin use, length z-score at birth, pregnancy induced hypertension (PIH), severe ICH, and feeding status at discharge, accelerated linear growth was associated with increased BSID-III cognitive score (+3.2 [0.02, 6.4], p=0.048), but not motor (+4.7 [−0.3, 9.8]) or language scores (+2.1 [−2.8, 7.0]), Figure 1. Weight and head circumference z-score change from birth to discharge were not associated with BSID-III cognitive, motor, or language scores.

When examining behavioral characteristics at two years, infants with weight and head circumference growth failure had significantly greater adjusted total M-CHAT-R score compared to those with normal weight growth (+0.43 [0.01,0.84], p=0.044 and +0.39 [0.04, 0.73], p=0.029). Infants with accelerated linear growth had a significantly lower adjusted total M-CHAT-R score compared to those with normal linear growth (−0.74 [−1.15, −0.34], p<0.01). There were no significant differences in CBCL scores in weight, length, or head circumference growth cohorts.

### Growth from Discharge to Two Years

Cohort characteristics are described for weight in Table 2 and length in **Supplementary Table 2**. Mothers of infants with accelerated weight and linear growth after discharge were more likely to have a high school education or less (Table 2 and **Supplementary Table 2**). Infants with weight growth failure after discharge were more likely to have had severe ICH and severe BPD (Table 2). Those with accelerated linear growth after discharge were likely to be lower gestational age at birth and small for gestational age (**Supplmentary Table 2**).

**Figure 2:**
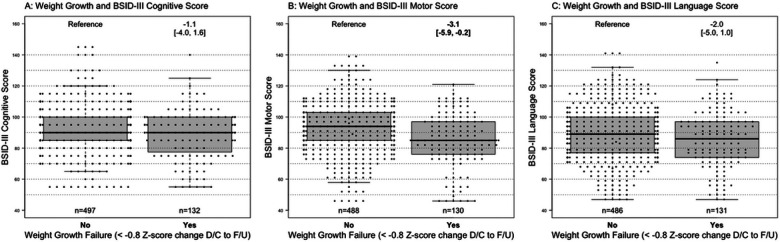
A-C: Weight growth failure from discharge (D/C) to two year follow-up (F/U) and Bayley Scales of Infant Development III (BSID-III) scores. Boxplots of BSID-III cognitive, motor, and language scores comparing infants who experienced weight growth failure from discharge to two year follow-up to those that did not, adjusting for gestational age, maternal education, >14 days of dexamethasone, erythropoietin use, length z-score at birth, pregnancy induced hypertension, severe bronchopulmonary dysplasia, and grade 3-4 intracranial hemorrhage. Estimated mean difference in the accelerate growth group with 95% CI is presented above the boxplot, with significant differences in bold.

After adjusting for gestational age, maternal education, >14 days of dexamethasone, erythropoietin use, length z-score at birth, PIH, severe ICH, and feeding status at discharge, weight growth failure at follow-up was associated with decreased motor (−3.1 [−5.9, −0.2], p=0.035) scores, but not language (−2.0 [−5.0, 1.0] or cognitive scores (−1.1 [−4.0, 1.6]), Figure 2. There were no differences in BSID-III cognitive, motor, and language scores with changes in length or head circumference z-score.

**Figure 3:**
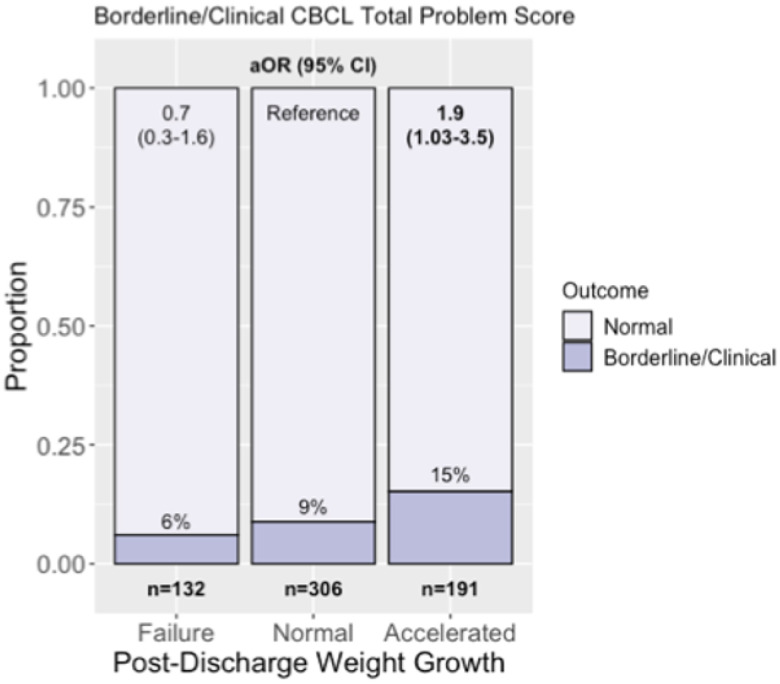
Total Behavioral Problems score and post-discharge weight growth. Odds of borderline/clinical Child Behavioral Checklist total problem score in infants with accelerated weight growth, weight growth failure, and normal weight growth from discharge to two years. Number within each group as well as proportion and percentage with borderline/clinical scores within each group is shown. Adjusted odds ratio (aOR) for borderline/clinical total problem score compared to those with normal growth is shown adjusted for gestational age, maternal education, >14 days of dexamethasone, erythropoietin use, length z-score at birth, pregnancy induced hypertension, severe bronchopulmonary dysplasia, and grade 3-4 intracranial hemorrhage.

When adjusting for gestational age, maternal education, >14 days of dexamethasone, erythropoietin use, length z-score at birth, PIH, severe BPD, feeding status at discharge, and severe ICH, infants with accelerated weight growth at two years had increased odds of total behavioral problems compared to those with normal growth and growth failure (OR 1.88 [1.03,3.45], p=0.041, Figure 3). Behavioral outcomes on the CBCL did not differ between linear or head circumference growth cohorts. There were no significant differences in M-CHAT-R scores.

## DISCUSSION

This large, multi-center study examined the association between growth parameter z-score changes to two year neurodevelopmental and behavioral outcomes among extremely premature infants. Utilizing contemporary definitions of growth failure currently endorsed by Academy of Nutrition and Dietetics (13), we did not find significant differences in BSID-III scores based on growth parameters. However, we found weight growth failure during initial hospitalization was associated with an increased M-CHAT-R score, more concerning for autism. In contrast, we found accelerated linear growth was associated with a modest increase in BSID-III cognitive score and decreased M-CHAT-R. Weight growth failure from discharge to two year follow-up was associated with modestly decreased motor score, and accelerated weight z-score gain was associated with increased total behavioral problems. These findings suggest the association of in-hospital growth failure with neurodevelopmental outcomes is not as robust as once believed, and potentially needs to be re-evaluated.

Studies examining growth during the NICU hospitalization and subsequent neurodevelopment are heterogenous and conflicting. Different metrics (velocities vs. changes in z-scores), differing time points of evaluation (birth vs. nadir, 36 weeks corrected age vs. discharge), and different growth curves (Fenton vs. INTERGROWTH) have been used. In this study, we utilized changes in z-scores utilizing the growth failure definition applied by Goldberg *et al*. using the Fenton growth curve from birth to discharge (13, 18). One study examining ELBW and very preterm infants utilizing these definitions did not find differences in neurodevelopmental outcomes on the BSID-II at 12 months (1). In contrast, one study found a decline in weight z-score > 1.2 to be associated with increased days to oral feeding (21), a known strong predictor of neurodevelopmental outcomes, and another found a weight z-score change > 1 to be associated with BSID-III scores (22). Other studies are similar to ours, identifying poor linear growth as detrimental to cognitive and language outcomes (8, 15, 23). These results call for standardized guidelines to be established prior to applying these definitions for clinical and quality improvement initiatives.

This study found weight growth failure from discharge to two years was associated with lower BSID-III motor scores. There is less research on growth following NICU discharge in extremely preterm infants. One study found that increase in head circumference z-score from discharge to follow-up was associated with improved mental processing composite score (24). Another large cohort study found weight velocity following discharge decreased the odds of neurodevelopmental impairment in a non-linear fashion (25). A study examining growth from discharge to two years found gains in weight/length z-score was associated with decreased odds of cognitive impairment at 10 years old (16). These studies suggest that growth following NICU discharge should be closely monitored by the pediatrician.

Although there are multiple studies showing preterm infants are at risk for behavioral and psychiatric problems, this is one of the few studies examining behavioral outcomes and growth trajectories during and after hospitalization (26-28). We found there were increased total behavioral problems with accelerated weight growth after discharge. One study found poor fetal growth *in utero* was associated with increased risk for attention-deficit hyperactivity disorder (29). Another recent study in extremely preterm infants did not find differences with different growth trajectories during NICU hospitalization and behavioral/psychiatric outcomes (16). Our study also found modest (< 1 point) differences in M-CHAT-R scores during NICU hospitalization. Accelerated linear growth during NICU hospitalization was associated with a decreased M-CHAT-R score and those with weight or head circumference growth failure during NICU hospitalization had an increased M-CHAT-R score. Of note, we used the M-CHAT-R. Previous research utilizing the original M-CHAT in ELBW infants found poor sensitivity and positive predictive value for autism (30). However, a recent study examining the M-CHAT-R and comparing that to the CBCL and the BSID in infants less than 30 weeks’ gestation found they were associated with neurobehavioral and CBCL outcomes, suggesting the revised M-CHAT may have utility as a developmental screen (31). The previously mentioned study and our study call for further research regarding extremely premature infants and autism screening.

One potential reason we did not appreciate substantial differences in neurodevelopmental outcomes in infants with weight growth failure during NICU hospitalization is due to the methodology of examining birth weight z-score rather than weight nadir z-score. The use of weight at birth versus nadir weight is currently controversial. Weight nadir z-score may better reflect expected physiologic weight loss (32). A study examining weight gain velocity calculations recommended the utilization of the weight nadir or day seven weight rather than birth weight (33). Day seven was deemed to not be significantly different than nadir and less laborious for clinicians and researchers (33). Our study chose to use birth weight z-score as this described in Goldberg *et al*. as this information is standardly available at majority of NICUs and thus is more generalizable.

We anticipate that linear growth affected cognitive and autism screening due to differences in lean body mass (bone, muscle, water). Compared to weight and head circumference, length is understood to be the anthropometric measure best associated with lean body mass/fat-free mass (34). Fat-free mass has been associated with improved neurodevelopmental outcomes. One study examining preterm infants found higher four month to four year fat-free mass gains were associated with improved full-scale IQ and speed of processing performance, whereas term to four month fat mass gains were associated with lower working memory (35). In infants with congenital gastrointestinal anomalies, higher fat free mass was associated with improved vocabulary, cognitive flexibility, and general cognitive function at preschool age (36). Another study examining extremely preterm infants found higher fat-free mass z-score was associated with larger brain size at term (37). Currently, body composition tools are deemed costly, and cannot be utilized in unstable infants. Further research involving use of low-cost tools such as ultrasound and bioimpedance are necessary to apply them in the clinical arena.

The strength of this secondary analysis is the utilization of a large, contemporary cohort of extremely premature infants across 30 NICUs in the United States (17). This study included a battery of developmental tests, including not only the use of BSID-III scores, but also CBCL and MCHAT-R scores (17). This allows for a more comprehensive developmental assessment. We also applied the use of anthropometric z-score assessments which are being adapted by many quality improvement networks in the United States; thus, our results are relevant to current clinical practice. Accounting for all of these factors, we believe these results are generalizable to other US-based NICUs.

This study also has limitations. It is a retrospective chart review, and nutritional management continues to evolve. Because the study was a multi-center randomized control trial for a different purpose, there is a lack of serial growth measurements during the hospital stay and post-discharge. Lengths were also performed per-unit protocol, and it is unclear if all sites were using length boards, which have been shown to be more accurate measures of an infant’s length (38). Because of the multi-center nature, there is not one standard parenteral and enteral nutritional protocol. We also do not have information on dietary intake after discharge.

In conclusion, extremely preterm infant growth trajectories during and after the NICU hospitalization showed modest associations with neurodevelopmental and behavioral outcomes. In-hospital linear growth appeared to be the most neuroprotective. These findings suggest that our current nutritional guidelines defining growth failure during NICU hospitalization may require further evaluation. Furthermore, nutritional studies exploring how to improve linear growth and how to measure lean body mass are imperative to improve neonatal nutrition in preterm infants. Also, further research is required on optimal growth trajectories after NICU hospitalization. Our study elicits further questions to be raised regarding the optimal metrics of adequate nutrition during hospitalization and following discharge in the extremely preterm infant.

## Figures and Tables

**Table 1.** Cohort subject characteristics during the timeframe of birth to hospital discharge. Growth failure and accelerated growth is defined as having length growth failure or accelerated length growth at discharge. Categorical variables are represented by n (%). Continuous variables are represented with mean (SD) or median (IQR). P-values were calculated with Chi-squared tests for categorical and ANOVA for continuous variables. Severe NEC was defined as Bell’s stage 2b to 3. Severe ICH was defined as grade III or IV either unilateral or bilateral, according to Papile staging. BPD was defined as requiring nasal canula or higher levels of respiratory support at 36 weeks PMA. Severe sepsis was defined as culture-proven bacterial or fungal sepsis resulting in blood-pressure support or substantive new respiratory support.

	Length Growth Failure, n (%) or median (IQR)	Normal Length Growth, n (%) or median (IQR)	Accelerated Length Growth, n (%) or median (IQR)	p-value
Included Infants (n=629 )	371	228	30	-
**Maternal Characteristics**				
Maternal age	28.6 (6.1)	29.8 (6.3)	30.7 (5.7)	**0.02**
Maternal education				
High School or less	133 (35.8)	58 (25.4)	7 (23.3)	**0.01**
Some College	102 (27.5)	82 (36.0)	10 (33.3)	0.06
Bachelor's or greater	87 (23.5)	70 (30.7)	10 (33.3)	0.06
Not reported	49 (13.2)	18 (7.9)	3 (10.0)	0.12
Pregnancy Induced Hypertension	31 (8.4)	18 (7.9)	1 (3.3)	0.36
Infant Characteristics				
Small for gestational age	49 (13.2)	28 (12.3)	3 (10.0)	0.55
Male Sex	191 (51.5)	114 (50.0)	15 (50.0)	0.71
Gestational Age	25.5 (1.1)	25.7 (1.1)	25.9 (1.0)	**<0.0001**
Birth Weight z-score	−0.0 (0.9)	−0.1 (0.9)	−0.1 (1.0)	0.33
Birth Length z-score	−0.2 (0.9)	−0.6 (0.9)	−1.5 (1.6)	**<0.0001**
Birth Head Circumference z-score	−0.3 (0.8)	−0.2 (0.9)	−0.1 (2.6)	0.68
Time to Regain Birth Weight	9 (6-12)	8 (5-12)	8 (7-11)	0.37
Discharge Weight z-score	−1.0 (0.8)	−0.7 (0.8)	−0.4 (0.9)	**<0.0001**
Discharge Length z-score	−2.1 (1.3)	−1.0 (1.3)	0.3 (1.3)	**<0.0001**
Discharge Head Circumference z-score	−0.9 (1.2)	−0.6 (1.0)	−0.2 (0.9)	**<0.0001**
Severe NEC	16 (4.3)	8 (3.5)	0 (0.0)	0.35
Severe ICH	41 (11.1)	24 (10.5)	4 (13.3)	0.46
Severe BPD	223 (60.1)	145 (63.6)	22 (73.3)	**0.04**
Severe Sepsis	23 (6.2)	9 (3.9)	1 (3.3)	0.27
>14 days of dexamethasone	25 (6.7)	18 (7.9)	1 (3.3)	0.46
Length of stay (days)	103 (32)	102 (30)	99 (20)	0.690
Weight Growth Velocity (g/day)	21.0 (4.3)	22.9 (4.5)	26.0 (4.0)	**<0.0001**
Length Growth Velocity (cm/week)	0.91 (0.2)	1.1 (0.1)	1.5 (0.3)	**<0.0001**

**Table 2.** Cohort comparisons during the timeframe of neonatal intensive care unit discharge to two year infant follow-up clinic. Growth failure and accelerated growth were categorized as having weight growth failure or accelerated weight growth from discharge to two year follow-up. Categorical variables are represented by n (%). Continuous variables are represented with mean (SD). P-values were calculated with Chi-squared tests for categorical and ANOVA for continuous variables. Severe ICH was defined as grade III or IV either unilateral or bilateral, according to Papile staging. BPD was defined as requiring nasal canula or higher levels of respiratory support at 36 weeks postmenstrual age.

	Weight Growth Failure, n (%)	Normal Weight Growth, n (%)	Accelerated Weight Growth n (%)	p-value
Included Infants	132	306	191	
Gestational Age, weeks	25.5 (1.1)	25.6 (1.1)	25.6 (1.1)	0.99
Birth weight z-score	−0.2 (1.0)	−0.1 (0.8)	−0.0 (0.8)	**0.047**
Birth length z-score	−0.6 (1.2)	−0.4 (1.0)	−0.3 (1.0)	0.06
Birth head circumference z-score	−0.2 (1.3)	−0.3 (1.0)	−0.3 (0.8)	0.50
Maternal education				
High School or less	31 (23.5)	87 (28.4)	80 (41.9)	**0.0009**
Some College	48 (36.4)	101 (33.0)	45 (23.6)	**0.01**
Bachelor's or greater	37 (28.0)	82 (26.8)	48 (25.1)	0.57
Not reported	16 (12.1)	36 (11.8)	18 (9.4)	0.44
SGA	24 (18.2)	38 (12.4)	18 (9.4)	**0.04**
PIH	11 (8.3)	18 (5.9)	21 (11.0)	0.33
GDM	11 (8.3)	12 (3.9)	10 (5.2)	0.36
Maternal obesity	8 (6.1)	35 (11.4)	20 (10.5)	0.22
Days to regain birth weight	8 (5-11)	9 (6-12)	9 (7-13)	**0.01**
Severe ICH	26 (19.7)	30 (9.8)	13 (6.8)	**0.0008**
Severe BPD	101 (76.5)	187 (61.1)	102 (53.4)	**<0.0001**

## Data Availability

The data that supports the findings of this study are available from the corresponding author upon reasonable request and may require institutional data agreements.
